# P-1700. A Ten-year study on Bacterial Identification using MALDI-TOF Mass Spectrometry in a Reference Laboratory

**DOI:** 10.1093/ofid/ofaf695.1872

**Published:** 2026-01-11

**Authors:** Danielle Wroblewski, Sai Laxmi Gubbala, Julia Connors, William J Wolfgang, Andrew Peifer, Reed Robinson, Ryan Bennett, Kimberlee A Musser, Lisa Mingle, Kara Mitchell

**Affiliations:** Wadsworth Center, New York State Department of Health, Albany, New York; New York State Department of Health: Wadsworth Center, Albany, New York; New York State Department of Health: Wadsworth Center, Albany, New York; New York State Department of Health: Wadsworth Center, Albany, New York; NYSDOH Wadsworth Center, Albany, New York; New York State Department of Health: Wadsworth Center, Albany, New York; New York State Department of Health: Wadsworth Center, Albany, New York; Wadsworth Center, New York State Department of Health, Albany, New York; Wadsworth Center, New York State Department of Health, Albany, New York; NY Wadsworth Lab, Albany, New York

## Abstract

**Background:**

Matrix assisted laser desorption/ionization time-of-flight mass spectrometry (MALDI- TOF MS) has been utilized for well over a decade for bacterial identification, especially in clinical laboratories. MALDI-TOF MS has become an invaluable tool in public health laboratories due to its rapid, accurate and inexpensive identification of bacteria. The Wadsworth Center Bacteriology Laboratory (WCBL) serves as the New York State public health reference laboratory for the identification of and characterization of bacterial pathogens of public health significance. In 2014, the WCBL validated Bruker Biotyper MALDI-TOF MS replacing 16S rRNA sequencing and biochemical testing for bacterial identification.

**Methods:**

The WCBL performed a ten-year review of the algorithms, identification rate, and turnaround time.

**Results:**

From 2015 to 2024, the WCBL received 112,952 specimens, ranging from a low of 6,603 specimens in 2015 to a high of 17,819 specimens in 2024. During this time, the WCBL tested more than 50,000 isolates by MALDI-TOF MS, ranging from 1708 isolates in 2015 to 9155 isolates in 2023. From 2015 to 2024, the WCBL was able to identify over 1,000 unique organisms, which aligned with manufacturer library improvements. During that same time frame, the top ten organisms identified each year changed slightly. The first several years, *Campylobacter jejuni* was the most identified organism, while in 2018, this shifted to *Pseudomonas aeruginosa*. This shift occurred when the WCBL became the Antimicrobial Resistance Laboratory Network (ARLN) regional laboratory for the Northeast. There was also a significant decrease in turnaround time for specimen reporting from a mean of over 9 days in 2013 prior to implementation versus ­­7 days in 2023 post implementation. This reduction in turnaround time occurred even with a large increase in specimen volume.

**Conclusion:**

This review displays the impact of MALDI-TOF MS implementation in a reference laboratory over a ten-year timeframe. The result of validating this method has had a significant impact, simplifying algorithms and decreasing turnaround times despite a large increase in sample burden.

**Disclosures:**

All Authors: No reported disclosures

